# News and Notes

**Published:** 1911-08

**Authors:** 


### NEWS *and* NOTES

### REGULAR MEETING FULTON COUNTY MEDICAL SOCIETY, AUGUST 3, 1911.

In discussing Dr. Blosser's paper on History of Medicine, a Neglected Subject, Dr. Geo. M. Niles expressed his appreciation of the manner in which this interesting subject was presented. He said that in the education of many of the doctors of to-day, reference to the past worthies of the profession, either real or mythological, was seldom indulged in, and that several years ago, when he told a physician in New York that he was a disciple of Aesculapius and a follower of Hippocrates, the statement was received with a blank look, showing that this learned medico had never heard of either of those wonderful characters.

Dr. Niles also indulged in a little mythological excursion, recounting how Aesculapius, in his way, had become so ambitious that he was not satisfied with curing ordinary mortals, but invaded the infernal regions, much to the displeasure of Pluto, who succeeded in having Jupiter slay Aesculapius with a shaft of lightning. Later on he was raised to the rank of god, while his lovely daughtr, Hygieia, was proclaimed the goddess of health, and a shrine was erected to them at Epidaurus on the coast of Laconica, where their praise were sung to the sound of the cornet the harp, the sackbut, the psaltery and the dulcimer.

Dr. Niles concluded by urging the study of the achievements of the many famous members of the healing art, men who played their part nobly, and have gone to their reward.

Dr. Holliday said that all through the social development of the world doctors have done a great many things worth while but there has been little or no acknowledgement of their efforts in general history. Wars and boundary changes have seemed more important than the saving of human life. Killing is more spectacular than the steady efforts to relieve pain and prevent disease ,and it has demanded more conspicuous and extensive recording.

The doctors in Georgia have established beyond a doubt that Crawford W. Long discovered the anaesthetic use of sulphuric ether, but even this comparatively recent event was almost lost from history in a maze of misinformation. Great though its benefits have been, our people have commemorated it only recently in monument to his memory. At Cornell University Dr. Long's picture is installed among those of celebrated men.

In Great Britain Sir James Y. Simpson has a monument as the first user of chloroform. In New York City there is a monument to Marion Lyons, but beyond these there is no recognition of the splendid successes, the magnificent sacrifices medical men have made. The discoveries and the victims of investigations in the work of establishing the nature of Malaria and Yellow Fever have had no enduring public recognition and the public has not been taught to realize the greatness of its debt to them. Cuch maters should have far more eminence assigned to them in public schools than such spectacular events as usually fill the books.

### DR. OSLER'S CHALLENGE TO THE ANTI-VACCINATIONISTS.

"A great deal of literature has been distributed, casting discredit upon the value of vaccination in the prevention of smallpox. I do not see how anyone who has gone through epidemics as I have, or who is familiar with the history of the subject, and who has any capacity left for clear judgment, can doubt its value. Some monhs ago I was twitted by the editor of the Journal of the Anti-Vaccination League for 'a curious silence' on this subject. I would like to issue a Mount Carmel-like challenge to any ten unvaccinated priests of Baal. I will go into the next severe epidemic with ten selected vaccinated persons and ten selected unvaccinated persons. I should prefer to choose the latter—three members of Parliament, three anti-vaccination doctors if they could be found, and four anti-vaccination propagandists. And I will make this promise—neither to jeer nor to jibe when they catch the disease, but to look after them as brothers, and for the four or five who are certain to die I will try to arrange the funerals with all the pomp and ceremony of an anti-vaccination demonstration."—*American Magazine.*

